# Dietary Astragalus Crude Extract Boosts Piglet Growth and Mitigates Weaning Stress by Enhancing Antioxidant Activity, Improving Immunity, and Modulating Intestinal Microbiota

**DOI:** 10.3390/vetsci13030242

**Published:** 2026-03-03

**Authors:** Yuyan Che, Long Cai, Longlong Zhu, Lu Li, Lufang Deng, Guoshun Chen, Jing Wang

**Affiliations:** 1 College of Animal Science and Technology, Gansu Agricultural University, Lanzhou 730070, China; 2 Institute of Animal Husbandry and Veterinary Medicine, Beijing Academy of Agriculture and Forestry Sciences, Beijing 100097, China; 3 Joint Laboratory of Animal Science between IAHVM of BAAFS and Division of Agricultural Science and Natural Resources of Oklahoma State University, Beijing 100097, China; 4 Department of Technology, Feed Branch of Beijing Sanyuan Breeding Technology Co., Ltd., Beijing 100163, China

**Keywords:** astragalus extract, antioxidant capacity, immune function, gut microbiota, weaned piglet

## Abstract

Astragalus is a traditional Chinese medicine that contains various bioactive compounds, which exert antioxidant and immunomodulatory properties. This research aimed to investigate the impacts of Astragalus crude extract on growth performance, nutrient digestibility, immune response, and intestinal health in weaned piglets. Sixty crossbred weaned piglets at 28 days of age (8.12 ± 0.14 kg) were allocated into three dietary treatments: a control group fed a corn–soybean meal-based diet (CON), an antibiotic group received the basal diet with 1000 mg/kg aureomycin (AN), and a treatment group administered the basal diet with 500 mg/kg Astragalus crude extract (CE), with 5 replicates per group, 4 piglets per replicate. The experimental trial lasted for 28 days. Feed intake and body weight were recorded to evaluate growth performance. Blood, intestine, and liver tissue samples were collected for the analysis of antioxidant capacity and gene expression. Compared to the CON group, supplementation with CE or AN significantly enhanced growth performance and decreased diarrhea incidence on days 0–14, whereas only CE tended to improve crude protein digestibility. Piglets supplemented with AN or CE showed improved antioxidant capacity and immune response relative to the CON group, as evidenced by increased liver and intestinal antioxidant enzyme activities, upregulated intestinal antioxidant gene expression, and enhanced plasma immunoglobulin A levels. Further, the upregulated intestinal tight junction protein expression was observed in CE or AN groups compared with the CON. Notably, dietary CE significantly modulated gut microbiota composition, particularly enriching the genera *Blautia*, *Turicibacter*, *Agathobacter*, and *Clostridium*. These findings indicate that dietary Astragalus crude extract promotes the gut health of weaned piglets by reducing oxidative stress and modulating gut microbiota composition, thereby decreasing diarrhea incidence and improving growth performance.

## Simple Summary

Weaning stress induces diarrhea and growth retardation in piglets. Astragalus has been demonstrated to exert bioactive properties, including antioxidant, anti-inflammatory, and immunomodulatory activities. This study investigated whether dietary supplementation with Astragalus crude extract could serve as a safe and effective alternative to antibiotics in weaned piglets. The results suggested that Astragalus crude extract supplementation mitigated weaning stress and improved the growth performance by enhancing antioxidant capacity, improving immune response, and modulating the microbiota composition in piglets. Its efficacy is comparable to that of antibiotics. This study provides a theoretical basis and practical support for the application of Astragalus to improve the intestinal health of weaned piglets.

## 1. Introduction

Weaning triggers intestinal redox imbalance, inflammatory responses, and increased intestinal permeability in piglets, ultimately resulting in weaning stress, which is a pathological condition characterized by anorexia, diarrhea, and growth retardation [1,2]. In recent decades, antibiotics have been widely used in livestock production to alleviate a series of diseases and poor production performance caused by weaning stress. However, their prolonged and excessive use has resulted in bacterial resistance and drug residues, which directly threaten food safety and human health [3,4]. Many countries have gradually prohibited the use of antibiotics as growth promoters in animal production in recent years. There is an urgent need to develop effective antibiotic alternatives to support growth performance and health status in animals. Chinese herbal medicine has attracted growing research attention owing to its wide sources, rich bioactive components, broad biological functions, and low risk of inducing drug resistance [5,6]. Among these herbs, Astragalus, as a food and medicinal homology, has attracted particular attention [7,8].

The major bioactive constituents responsible for the primary efficacy of Astragalus extracts include polysaccharides, saponins, and flavonoids; these compounds exhibit antioxidant, anti-inflammatory, and immunomodulatory properties [8,9]. Astragalus polysaccharides have been demonstrated to enhance Immunoglobulin G (IgG) and Immunoglobulin M (IgM) in sow colostrum and increase maternal milk IgA levels [10,11]. They also decrease Interleukin 1β (IL-1β) and tumor necrosis factor α (TNF-α) levels while boosting superoxide dismutase (SOD) and total antioxidant activity (T-AOC) activity in lipopolysaccharide-challenged piglets [12]. Astragaloside IV, a cycloartane-type triterpenoid saponin and one of the major bioactive constituents of Astragalus, has been shown to enhance innate immune function by improving the antigen presentation function of porcine alveolar macrophages, promoting the expression of nuclear factor erythroid 2-related factor 2 (Nrf2) and heme oxygenase (HO-1), and restoring the levels of SOD and catalase (CAT) in mice of dextran sulfate sodium (DSS)-induced colitis [13,14]. In addition, our previous study also found that calycosin, an isoflavone derived from Astragalus, could attenuate the H_2_O_2_-induced reduction in CAT and T-AOC activity of porcine intestinal epithelial cells [15]. However, most current studies have focused on Astragalus individual components or their combinations with other herbal extracts, whereas relatively few reports have investigated the effects of supplementing with Astragalus crude extract alone in piglets. In practical livestock production, crude extracts are more commonly used due to their cost-effectiveness, ease of preparation, and potential interaction effects among multiple constituents. Traditional herbal medicine theory also proposes that the therapeutic effects of medicinal herbs may result from the coordinated actions of multiple bioactive constituents rather than from a single isolated compound [16]. Therefore, investigating the integrated effects of Astragalus crude extract is of both scientific and practical significance.

Given that individual active constituents from Astragalus have already been shown to alleviate oxidative stress and intestinal inflammation, we hypothesize that Astragalus crude extract exerts considerable potential for mitigating weaning stress and improving the intestinal health of weaned piglets through the integrated effects of its various active components. Accordingly, the present study aims to systematically assess the effects of Astragalus crude extract supplementation on growth performance and incidence of diarrhea in weaned piglets, and to further elucidate the underlying beneficial mechanisms through comprehensive evaluations of antioxidant capacity, intestinal barrier function, and gut microbiota composition. These findings are expected to provide a scientific basis for the utilization of Astragalus crude extract as a safe and effective alternative to antibiotics in piglet production.

## 2. Materials and Methods

### 2.1. Study Design and Dietary Interventions

Sixty crossbred Duroc × (Landrace × Yorkshire) piglets weaned at 28 d with an initial body weight (BW) of 8.12 ± 0.14 kg were randomly divided into three dietary treatments: a control group fed basal diet without antibiotic (CON), an antibiotic group received the basal diet with 1000 mg/kg aureomycin (AN), and a treatment group administered the basal diet with 500 mg/kg Astragalus crude extract (CE), with 5 replicates per group, 4 piglets (equal numbers of males and females) per replicate. Piglets were group-housed at a density of four piglets per pen featuring a flooring design with one-third slatted area, maintained at 25–28 °C and 50–60% relative humidity. The experiment was conducted over 28 days and comprised two distinct phases: 0–14 days and 15–28 days. Feed intake and BW were recorded to evaluate growth performance. Astragalus crude extract was added to the basal diet step by step. The basal diet (a corn–soybean meal) was formulated to meet the nutritional requirements under the nutritional guidelines, following the guidelines established by the National Research Council (2012). The doses of Astragalus crude extract used in this experiment were based on previous reports and manufacturer recommendations [17]. The complete details of diet composition, including ingredients and nutrient profiles, are presented in Table 1. The Astragalus crude extract (10:1) was obtained from Chengdu KingTiger, containing 56.37% polysaccharides, 3.72% total saponins, 1.91% total flavonoids, along with minor amounts of amino acids and trace elements.

### 2.2. Sample Collection

Three days prior to termination of the trial, fresh fecal and feed samples were collected from each treatment and then pooled. Approximately 300–400 g of the composite fecal sample was mixed with 20% sulfuric acid and then stored at −20 °C until further analysis. Sulfuric acid was added to prevent nitrogen loss during storage and subsequent drying. The samples were then dried at 65 °C for 72 h to prepare them for subsequent analysis of nutrient digestibility. After 12 h of fasting on the 14th day of the trial, venous blood (10 mL per piglet) was drawn from the anterior vena cava by puncture, followed by centrifugation at 4 °C to separate plasma. Following blood sampling, one piglet per pen was humanely euthanized by electrical stunning followed by exsanguination. The electrical parameters used were: voltage 200–300 V, frequency 50–60 Hz, and a duration of at least 3 s [18]. Ileal digesta were collected for 16s rRNA gene sequencing analysis. Segments of jejunum and ileum were longitudinally opened with sterile surgical scissors, rinsed thoroughly with ice-cold saline to clear luminal contents, and the mucosal layer was scraped using sterile glass slides. The scraped mucosa was then snap-frozen in liquid nitrogen for later analysis. Approximately 50 g of liver tissues were collected and kept at −80 °C until analysis.

### 2.3. Growth Performance, Diarrhea Rate, and Nutrient Digestibility

BW was measured on days 0, 14, and 28 for the calculation of average daily gain (ADG). Meanwhile, feed intake was monitored daily per replicate to assess average daily feed intake (ADFI). During days 1–14, the fecal score was monitored through visual assessment (3, well-formed feces; 2, normal feces; 1, sloppy feces; 0, diarrhea). The calculation method of fecal score and diarrhea was referred to the previous study [19].

The apparent total tract digestibility (ATTD) of nutrients was determined using acid-insoluble ash (AIA) as an internal marker. Briefly, approximately 2 g of feed and fecal sample was processed following the standard ash determination protocol until constant weight was attained. Subsequently, 15–25 mL of hydrochloric acid solution was added, boiled for 10 min, and cooled. After evaporating moisture via water bath, the residue was incinerated at 550 °C for 1 h in a muffle furnace. Finally, it was cooled in a desiccator and weighed until constant mass was achieved. AIA content = (AIA mass/sample mass) ×100%. The Kjeldahl method (AOAC 984.13) was employed to determine the crude protein (CP) content, where CP (%) = total nitrogen content (%) × 6.25; ether extract (EE) content was determined using Soxhlet extraction according to AOAC method 920.39; the dry matter (DM) content was measured through oven drying at a temperature of 105 °C (AOAC 934.01) [20]. The ATTD was calculated by the formula below:

$$\mathrm{ATTD}\;(\%)=[1-\frac{\mathrm{nutrient}\;\mathrm{concentration}\;\mathrm{in}\;\mathrm{feces}\times \mathrm{AIA}\;\mathrm{concentration}\;\mathrm{in}\;\mathrm{diet}}{\mathrm{nutrient}\;\mathrm{concentration}\;\mathrm{in}\;\mathrm{diet}\times \mathrm{AIA}\;\mathrm{concentration}\;\mathrm{in}\;\mathrm{feces}}]\times 100$$

### 2.4. Plasma Parameters

Plasma samples were thawed at 4 °C and centrifuged for biochemical analysis. Plasma biochemical parameters were quantified using an automatic biochemical analyzer (Chemray 800, Rayto Life Technology, Shenzhen, China). The analyzed parameters included plasma liver enzyme profiles of alanine aminotransferase (ALT), alkaline phosphatase (ALP), and aspartate aminotransferase (AST) activity; lipid metabolism parameters of total cholesterol (TC), high-density lipoprotein cholesterol (HDL-C), low-density lipoprotein cholesterol (LDL-C), triglyceride (TG); and protein metabolic of blood urea nitrogen (BUN), total protein (TP) and albumin (ALB).

### 2.5. Enzyme-Linked Immunosorbent Assay

Liver and intestinal mucosa samples were homogenized mechanically in ice-cold phosphate-buffered saline using a refrigerated homogenizer. The resulting homogenate was then clarified by centrifugation (12,000× *g*, 15 min, 4 °C) to collect the supernatants. Total protein concentration was quantified with a commercial bicinchoninic acid assay kit (BCA, Huaxingbio, Beijing, China), following the manufacturer’s protocol. The plasma levels of IgA, IgG, and IgM (cat: YJ-98524, YJ42369, and YJ-12658), IL-1β, IL-6, and IL-10, and TNF-α (cat: YJ022366, YJ663251, YJ027436, and YJ 002360) were quantified using commercial enzyme-linked immunosorbent assay (ELISA) kits (Shanghai Yuanju, Shanghai, China); plasma diamine oxidase (DAO, cat: DKM2578B) activity was measured using commercial kits from Beijing Dakome Technology (Beijing, China). Quality control assessments verified that all ELISA procedures maintained less than 12% variation for both intra-assay and inter-assay measurements.

The T-AOC (cat: A015-3-1) within the samples was assessed utilizing the ferric reducing antioxidant power assay (Nanjing Jiancheng Bioengineering Research Institute, Jiangsu, China). In this method, antioxidants reduce Fe^3+^-tripyridyltriazine to its blue-colored Fe^2+^ form under acidic conditions. The T-AOC was quantified by absorbance measurement at 593 nm. Total SOD (T-SOD, cat: S0101) activity was determined using a WST-8-based assay (Beyotime Biotechnology, Shanghai, China). In this system, superoxide anions produced by xanthine oxidase react with WST-8 to generate a water-soluble formazan dye. The SOD activity was quantified by absorbance measurement at 450 nm. Using the thiobarbituric acid (TBA) assay to determine malondialdehyde (MDA, Beyotime Biotechnology, Shanghai, China) concentration (cat: S0131). The MDA content was determined via its reaction with TBA under acidic conditions at 90–100 °C to generate a red-colored adduct. The absorbance of the resulting product was subsequently measured at 535 nm, following the standard assay protocol.

### 2.6. RNA Extraction and RT-qPCR Quantification

Extraction and purification of total RNA from liver and intestinal tissue samples were performed with RNA-zol (Molecular Research Center, Cincinnati, OH, USA) as recommended by the supplier. RNA concentration was quantified and its quality assessed employing a DeNovix DS-11 ultra-microvolume spectrophotometer (DeNovix, Wilmington, DE, USA). Subsequently, cDNA synthesis was performed on the isolated RNA samples using a commercial reverse transcription kit (Bio-Rad, Hercules, CA, USA) under the manufacturer’s instructions. RT-qPCR was carried out using SYBR Green Supermix (Bio-Rad, Hercules, CA, USA). The reaction system composition was based on our previous study [19]. The primer sequences utilized in this study were designed with the Primer-Blast tool from NCBI. The specific sequences are detailed in Supplementary Table S1, and all primers were synthesized by Sangon Biotech (Shanghai, China). The relative quantification of gene expression was performed using the 2^−∆∆Ct^ method, with GAPDH serving as the housekeeping gene.

### 2.7. Western Blot Analysis

Total proteins were isolated from the intestine using a radioimmunoprecipitation assay buffer (cat: R1091, LABLEAD, Beijing, China), and protein concentrations were subsequently determined through BCA assays. Approximately 40–50 μg of the target protein was separated using 12% SDS-PAGE (cat: 1610185, Bio-Rad, Hercules, CA, USA), subsequently transferred onto PVDF membranes for Western blot analysis. After blocking for 30 min at room temperature (RT) using a commercial blocking buffer (cat: P0252, Beyotime Biotechnology, Shanghai, China), the membranes were then probed with the primary antibody at 4 °C overnight under continuous shaking. The primary polyclonal antibodies against Occludin (1:1000, cat: A2601, Abclonal), CLDN1 (1:1000, cat: bs-0790R) (Bioss, Beijing, China), and GAPDH (1:5000, cat: 6004-1-Ig) (Proteintech, Wuhan, China) were used in this study. Membranes were then incubated at RT for 1 h with goat anti-rabbit or anti-mouse IgG antibodies (1:5000 dilutions, Dakome). Chemiluminescent signals were obtained with the ChemiDoc™ Imaging System (Bio-Rad, Hercules, CA, USA).

### 2.8. 16s rRNA Analysis of Ileal Digesta

Genomic DNA was isolated from ileal digesta samples as previously described [19]. The V3–V4 hypervariable regions of the bacterial 16S rRNA gene were PCR-amplified and sequenced by Majorbio Bio-Pharm Technology (Shanghai, China) using a high-throughput sequencing platform. After initial quality control, sequencing reads were denoised using DADA2 workflow to generate amplicon sequence variants (ASVs). Taxonomic annotation of ASVs was carried out by comparison with the SILVA 16S rRNA gene reference database. Microbial diversity metrics, including alpha and beta diversity, were calculated using Mothur (version 1.30.2), with subsequent visualizations presented in R software (version 3.3.1). Community dissimilarities among experimental groups were evaluated based on Bray–Curtis distances and visualized by principal coordinate analysis (pCoA), with statistical significance assessed using ANOSIM. The Wilcoxon test was applied to identify genera showing differential relative abundance between groups. Correlations between the relative abundances of the 30 most prevalent taxa and antioxidant enzyme activities in plasma or intestinal tissues were evaluated using Spearman’s correlation analysis, and the results were visualized using pheatmap.

### 2.9. Statistical Analysis

Statistical analysis was conducted using SPSS (v26.0, IBM, Chicago, IL, USA). After confirming normal distribution, group comparisons were performed via one-way ANOVA, followed by pairwise comparisons with the least significant difference (LSD) post hoc test when significant effects were detected. The pen served as the experimental unit in all ANOVA analyses. Data that were not normally distributed were analyzed using the Kruskal–Wallis test. Results are presented as mean ± SEM. GraphPad Prism (v8.0.2; CA, USA) was used to generate bar graphs, with significance set at *p* < 0.05 and 0.05 ≤ *p* < 0.10 considered a trend.

## 3. Results

### 3.1. CE Modulates Growth and Nutrient Digestibility in Piglets

As indicated in Table 2, CE or AN supplementation significantly increased BW at day 14 and day 28 compared with the CON group (*p* < 0.01). Consistently, the ADG of piglets in the CE and AN group was markedly higher than that of the CON group during days 0–14 and the overall period (days 0–28) (*p* < 0.01). Moreover, AN supplementation significantly increased ADG on days 15–28 compared to the CON group (*p* < 0.05). In terms of feed intake, piglet supplementation with CE or AN significantly enhanced ADFI during days 15–28 and days 0–28 compared to the CON group (*p* < 0.01). And the feed-to-gain ratio (F/G) was significantly reduced in the CE and AN groups during days 0–14 compared with the CON group (*p* < 0.01), suggesting a comparable feed efficiency between CE and AN in the early weaning stage. No significant differences were observed among the groups during days 15–28 and the overall period (days 0–28) (*p* > 0.01).

Regarding diarrhea-related parameters, the CE and AN group exhibited a significantly lower diarrhea rate during days 0–14 compared with the CON group (*p* < 0.05). Similarly, the fecal score was significantly reduced in the AN group compared to the CON group (*p* < 0.05), while no significant difference was observed between the AN and CE group (*p* > 0.05) (Table 2).

As presented in Table 3, compared to the CON group, CE supplementation tended to improve CP digestibility (*p* = 0.083). AN supplementation significantly increased EE digestibility compared to the CON group (*p* < 0.05), while no significant difference was observed between the AN and CE groups. No significant differences in DM digestibility were observed among groups (*p* > 0.05).

### 3.2. CE Affects Plasma Biochemistry in Piglets

Compared to the CON group, plasma biochemical analysis showed that AN (*p* < 0.05) or CE (*p* = 0.089) supplementation decreased BUN levels. Piglets in the AN group showed significantly increased ALB concentration compared to the CON and CE groups (*p* < 0.05).

In terms of lipid metabolism, piglets in the CE or AN group showed significantly lower concentrations of TC and LDL-C compared with the CON group (*p* < 0.05). In addition, plasma levels of TP, HDL-C, TG, AST, ALT, and ALP did not show significant differences among the experimental groups (*p* > 0.05) (Table 4).

### 3.3. CE Modulates Immune and Cytokine Responses in Piglets

**Figure 1:**
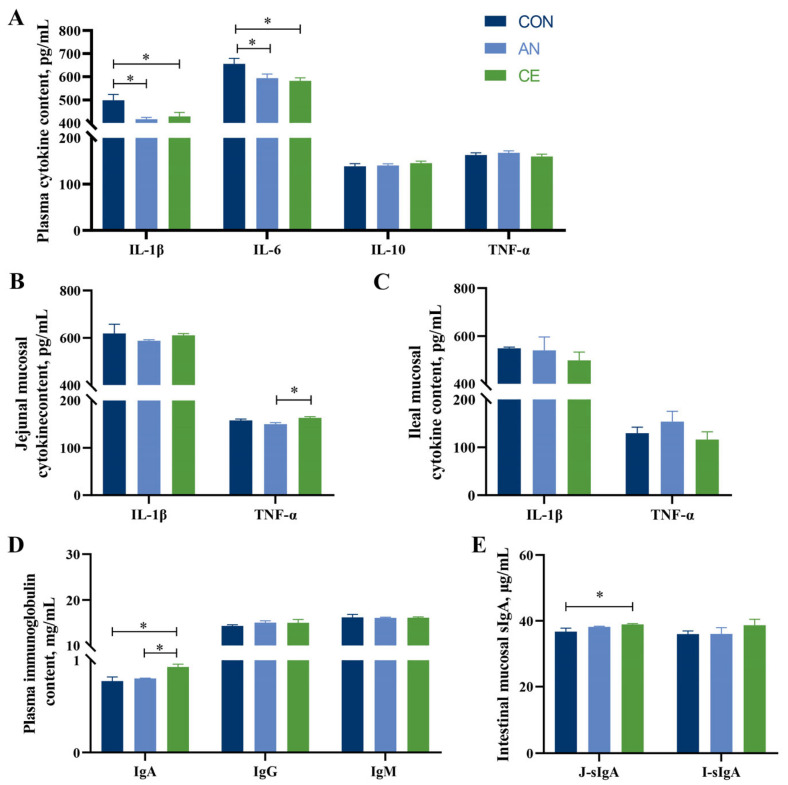
Effects of AN or CE on plasma and intestinal mucosal cytokine and immunoglobulin levels in piglets at 14 days post-weaning. (**A**) Plasma cytokine content of IL-1β, IL-6, IL-10, and TNF-α (**B**) Jejunal mucosal cytokine content of IL-1β and TNF-α (**C**) Ileal mucosal cytokine content of IL-1β and TNF-α (**D**) Plasma IgA, G, and M content (**E**) Jejunal and ileal mucosal secretory IgA (sIgA) content. CON, a control fed the basal diet; AN, the basal diet with 1000 mg/kg aureomycin; CE, the basal diet containing 500 mg/kg Astragalus crude extract. IL-1β, interleukin 1β; IL-6, interleukin 6; IL-10, interleukin 10; TNF-α, tumor necrosis factor α; IgA, IgG, IgM, immunoglobulin A, G, M. SEM, standard error of the means. The data are expressed as mean ± SEM, *n* = 5 in each group. * *p* < 0.05.

Compared to the CON group, AN or CE supplementation similarly reduced plasma levels of IL-1β and IL-6 (*p* < 0.05, Figure 1A). The CE group demonstrated significantly higher plasma IgA concentrations than both the CON and AN groups (*p* < 0.05, Figure 1D). Jejunal mucosa secretory IgA (sIgA) was significantly increased in the CE group (*p* < 0.05, Figure 1E) compared to the CON group (*p* > 0.05). Plasma IL-10, TNF-α, IgG, IgM levels, and ileal sIgA concentration exhibited no differences across the three groups (*p* > 0.05) (Figure 1A–E).

### 3.4. CE Modulates Plasma and Tissues Antioxidant Status in Piglets

**Figure 2:**
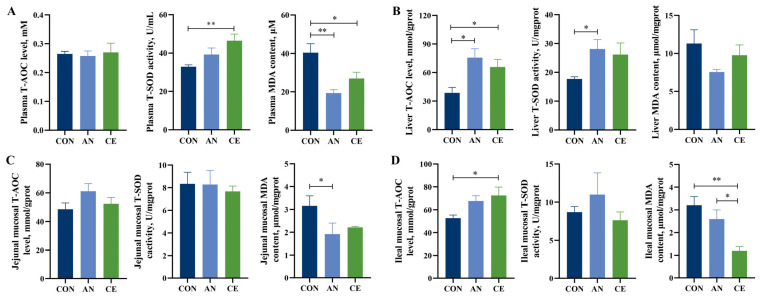
Effect of AN or CE on plasma, liver, and intestinal mucosa antioxidant enzyme activities in piglets at 14 days post-weaning. (**A**) Plasma T-AOC level, T-SOD activity, and MDA content (**B**) Liver T-AOC level, T-SOD activity, and MDA content (**C**) Jejunal mucosal T-AOC level, T-SOD activity, and MDA content (**D**) Ileal mucosal T-AOC level, T-SOD activity, and MDA content. CON, a control fed the basal diet; AN, the basal diet with 1000 mg/kg aureomycin; CE, the basal diet containing 500 mg/kg Astragalus crude extract. T-AOC, total antioxidant capacity; T-SOD, total superoxide dismutase; MDA, malondialdehyde. SEM, standard error of the means. The data are expressed as mean ± SEM, *n* = 5 in each group. * *p* < 0.05, ** *p* < 0.01.

The results of the antioxidant parameters are shown in Figure 2. Both AN (*p* < 0.01) and CE (*p* < 0.05) supplementation significantly reduced plasma MDA concentrations compared to the CON group (Figure 2A). Compared to the CON group, CE administration resulted in a significant elevation of T-SOD activity in plasma (*p* < 0.05); no significant difference was observed between the CE and AN groups (*p* > 0.05, Figure 2A). Dietary AN or CE supplementation significantly enhanced hepatic T-AOC levels; only AN supplementation increased T-SOD activity compared with CON group (*p* < 0.05, Figure 2B). Jejunal mucosa MDA content was significantly decreased in the AN group compared to the CON group (*p* < 0.05) (Figure 2C). Notably, dietary CE supplementation significantly increased ileal T-AOC levels compared to the CON. Ileal MDA content was significantly decreased in the CE group compared with both AN (*p* < 0.05) and CON (*p* < 0.01) groups (Figure 2D). There was no difference in plasma T-AOC, jejunal T-AOC, and T-S OD level, and ileal T-SOD activity among all groups (*p* > 0.05) (Figure 2A–D).

### 3.5. CE Regulates Antioxidant-Related Gene Expression of Piglets

**Figure 3:**
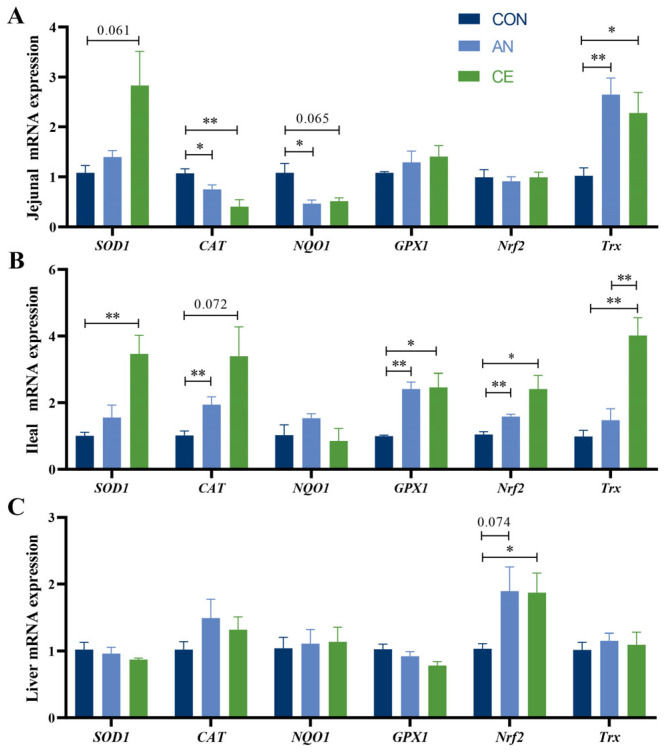
Effect of AN or CE on relative mRNA expression levels of the liver and intestinal tract in piglets at 14 days post-weaning. (**A**) Jejunal mRNA expression (**B**) Ileal mRNA expression (**C**) Liver antioxidant mRNA expression. CON, a control fed the basal diet; AN, the basal diet with 1000 mg/kg aureomycin; CE, the basal diet containing 500 mg/kg Astragalus crude extract. *SOD1*, superoxide dismutase 1; *CAT*, catalase; *NQO1*, NAD (P)H quinone oxidoreductase 1; *HO-1*, Heme Oxygenase 1; *GPX1*, glutathione peroxidase 1; *Nrf2*, nuclear factor erythroid 2-related factor 2; *Trx*, thioredoxin. SEM, standard error of the means. The data are expressed as mean ± SEM, *n* = 5 in each group. * *p* < 0.05, ** *p* < 0.01.

In jejunal tissues, compared to the CON group, CE supplementation upregulated *Trx* (*p* < 0.05) and *SOD1* (*p* = 0.061) mRNA expression while downregulating catalase (*CAT*) (*p* < 0.01) mRNA and *NQO1* (*p* = 0.065) expression. Dietary supplementation with AN upregulated *Trx* mRNA (*p* < 0.01) expression and downregulated *NQO1* and *CAT* expression (*p* < 0.05), whereas no differences in *GPX1* and *Nrf2* expression were detected across the groups. (*p* > 0.05) (Figure 3A). There’s no significant difference observed between CE and AN groups (*p* > 0.05). In ileal tissues, compared to the CON group, both AN (*p* < 0.01) and CE (*p* < 0.05) administration markedly enhanced *GPX1* and *Nrf2* expression. *CAT* expression was significantly increased in the AN group and tended to increase *CAT* expression in the CE group relative to the CON group (*p* = 0.072). Specifically, CE supplementation markedly elevated *SOD1* and *Trx* levels (*p* < 0.01). *Trx* mRNA level was upregulated by CE supplementation compared with the AN group (*p* < 0.01) (Figure 3B). Moreover, the hepatic *Nrf2* mRNA level in the AN and CE groups was increased (*p* = 0.074, *p* < 0.05) relative to the CON group. Other hepatic antioxidant-related gene expression remained unaltered across all experimental groups (Figure 3C).

### 3.6. CE Modulates the Intestinal Barrier Function in Piglets

**Figure 4:**
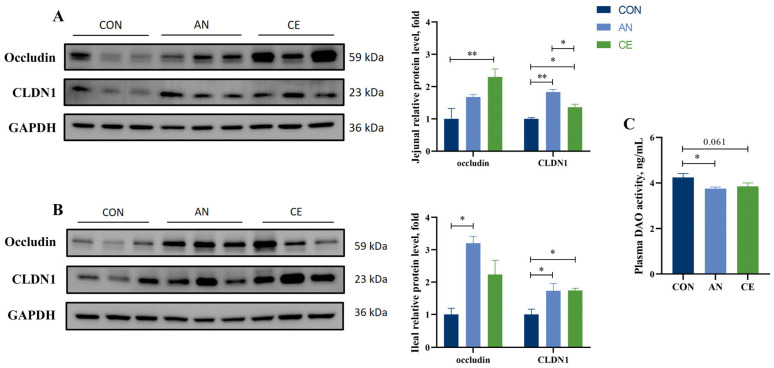
Effect of AN or CE on intestinal barrier function in piglets at 14 days post-weaning. (**A**) Western blot of jejunal tight junction protein and relative protein level (**B**) Western blot of ileal tight junction protein and relative protein level (**C**) Plasma DAO activity of weaned piglet. CON, a control fed the basal diet; AN, the basal diet with 1000 mg/kg aureomycin; CE, the basal diet containing 500 mg/kg Astragalus crude extract. DAO, diamine oxidase. SEM, standard error of the means. The data are expressed as mean ± SEM, DAO activity determination: *n* = 5 in each group; Western blot of tight junction protein: *n* = 3. * *p* < 0.05, ** *p* < 0.01.

As illustrated in Figure 4A,B, both AN and CE supplementation significantly enhanced intestinal epithelial barrier integrity in piglets. Compared to the CON group, AN or CE administration markedly upregulated CLDN1 protein expression in both jejunal and ileal tissues (*p* < 0.05). Specifically, CE supplementation significantly elevated jejunal occludin levels (*p* < 0.01), while AN treatment increased ileal occludin expression compared to the CON group (*p* < 0.05). Moreover, the reduction in DAO activity was observed in the AN (*p* < 0.05) and CE (*p* = 0.061) groups compared to the CON group, while no significant difference was observed between AN and CE groups (*p* > 0.05) (Figure 4C).

### 3.7. Effect of CE on Ileal Microbial Diversity and Composition in Piglets

**Figure 5:**
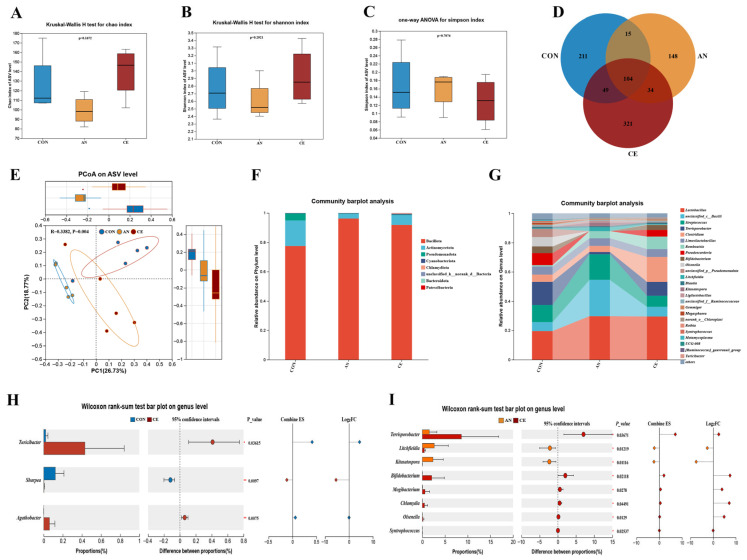
Effect of AN or CE on ileal microbiota in piglets at 14 days post-weaning. (**A**–**C**) α-diversity of Chao, Shannon, and Simpson index (**D**) Venn diagram of the amplicon sequence variant counts (**E**) β-diversity of principal coordinate analysis (PCoA) (**F**,**G**) Microbial composition at phylum and genus level (**H**) Genus-level differential analysis between CON and CE groups (**I**) Genus-level differential analysis between AN and CE groups. CON, a control fed the basal diet; AN, the basal diet with 1000 mg/kg aureomycin; CE, the basal diet containing 500 mg/kg Astragalus crude extract.

Dietary supplementation with CE significantly altered β-diversity of the ileal microbiota, as indicated by pCoA at the amplicon sequence variant (ASV) level. Significant separation of microbial community structures was observed among all groups (*p* = 0.004, R^2^ = 0.338). Venn diagram analysis showed that the CE group exhibited the highest richness of unique ASVs (321), compared to 211 in the CON group and 148 in the AN group. Statistical analysis indicated that α-diversity did not differ significantly among all groups (*p* > 0.05) (Figure 5A–E). The four dominant phyla were Bacillota, Actinobacteriota, Pseudomonadota, and Cyanobacteria in phylum level. Bacillota exhibited a significantly greater relative abundance in the AN and CE groups than in the CON group. At the genus level, *Lactobacillus*, *unclassified_c__Bacilli*, *Streptococcus*, *Terrisporobacter*, *Clostridium*, *Limosilactobacillus*, *Romboutsia*, *Pseudoscardovia*, *Bifidobacterium* and *Olsenella* was the dominant taxa (Figure 5G). Genus-level differential analysis using the Wilcoxon rank-sum test indicated a significant enrichment of *Turicibacter* and *Agathobacter* in the CE group relative to the CON group (Figure 5H). When compared to the AN group, the CE group exhibited significant enrichment of *Terrisporobacter* and *Bifidobacteriaceae*, while *Litchfieldia* and *Kitasatospora* genera were enriched in the AN group (Figure 5I).

### 3.8. CE Supplementation Mediates Oxidative Stress Attenuation *via* Gut Microbiota Modulation in Piglets

**Figure 6:**
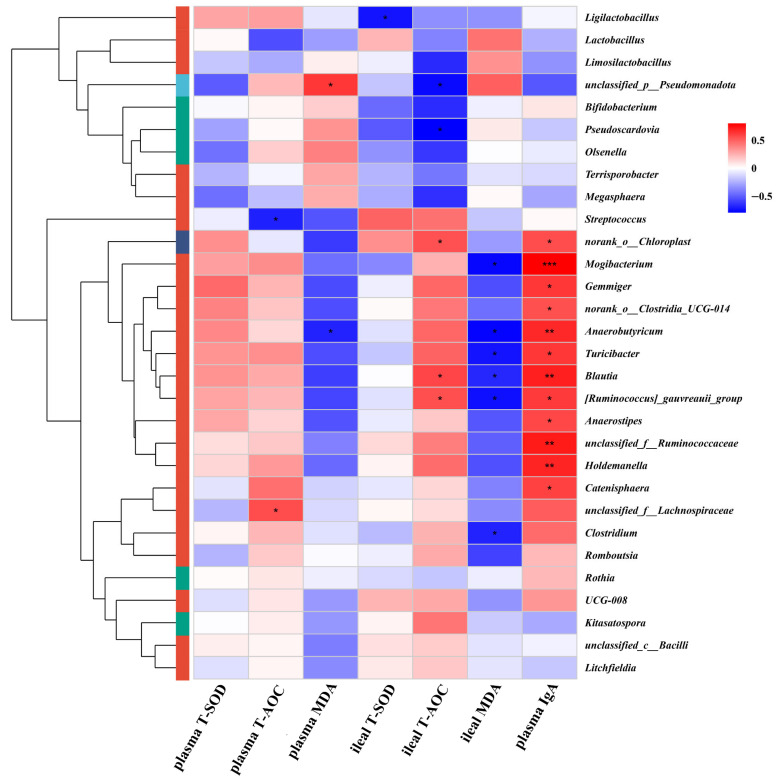
Correlation heatmaps between plasma antioxidant enzyme activity and bacterial genera. T-SOD: total superoxide dismutase; T-AOC: total antioxidant activity; MDA: malondialdehyde; IgA: immunoglobulin A. * 0.01< *p* ≤ 0.05, ** 0.001 < *p* ≤ 0.01, *** *p* ≤ 0.001.

The correlation heatmap between the abundant ileal microbiota genera in piglets and the oxidative stress indices and IgA in both plasma and ileum is presented in Figure 6. A negative correlation was observed between ileal MDA levels and the genera *Anaerobutyricum*, *Turicibacter*, *Blautia*, *Mogibacterium*, and *Clostridium* (*p* < 0.05). Ileal T-AOC was positively correlated with *norank_o__Chloroplast*, *Blautia*, and *[Ruminococcus]_gauvreauii_group* (*p* < 0.05). Plasma T-AOC showed a positive association *unclassified_f__Lachnospiraceae*, while it was negatively correlated with *Streptococcus* (*p* < 0.05). Moreover, like *Turicibacter*, *Blautia*, *Anaerobutyricum*, *Anaerostipes*, *Holdemanella*, *[Ruminococcus]_gauvreauii_group, unclassified_f__Ruminococcaceae*, and *Catenisphaera* beneficial taxa were significantly positively correlated with plasma IgA levels (*p* < 0.05, *p* < 0.01).

## 4. Discussion

In recent years, a wide range of herbs and other phytogenic products have been increasingly used as alternatives to antibiotics because of their recognized benefits in enhancing growth performance and intestinal health [21]. A previous study showed that Astragalus polysaccharides enhanced ADG and feed conversion ratio (FCR), as well as reduced the BUN level of piglets challenged with lipopolysaccharide [12]. Similarly, dietary Astragalus dregs supplementation was reported to improve ADG and ADFI of fattening pigs [22]. Similar results were observed in this study; dietary supplementation with Astragalus crude extract during the first 14 days post-weaning enhanced growth performance, as evidenced by increasing ADG, ADFI, and FCR. The observed enhancement in feed intake may be attributed to the inherent sweetness of polysaccharides and other palatability-enhancing components present in the extract, which could potentially exert appetite-stimulating effects [23]. Furthermore, the increase in CP digestibility and the decrease in BUN level observed in this study indicate that the addition of Astragalus crude extract may facilitate protein deposition, thereby contributing to improved growth performance of weaned piglets [24]. Taken together, these findings suggest that Astragalus crude extract enhances ADFI and nutrient utilization efficiency in weaned piglets, ultimately supporting better growth performance.

Weaning stress commonly triggers gastrointestinal infection and excessive inflammatory responses, often leading to diarrhea in piglets [25]. In the present study, dietary supplementation with Astragalus crude extract significantly decreased diarrhea incidence, an effect comparable to that observed in the antibiotic-treated group. This finding aligns with previous reports demonstrating that supplementation with other herbal extracts, such as *Cortex Phellodendri* extract, also attenuated piglet diarrhea during the weaning stage [26]. Additionally, Astragalus extract administration reduced plasma IL-1β and IL-6 levels, along with an increased IgA level. Consistently, a previous study demonstrated that Astragalus polysaccharides were shown to attenuate PRRSV-induced inflammatory responses by suppressing the expression of *TLR7*, *TLR9*, and *TNF-α* [27]. Our previous study has also shown that flavonoids from Astragalus effectively reduce pro-inflammatory cytokine levels while upregulating intestinal host defense peptide expression, thereby strengthening mucosal immune defense [19]. In addition, combined supplementation of Astragalus and *Codonopsis Pilosulae* has been reported to markedly increase serum IgM, IgG, and IgA concentrations in weaned piglets [28]. Taken together, these findings suggest that the reduction in diarrhea incidence observed in the present study may be mechanistically associated with the attenuation of excessive inflammatory responses and the restoration of intestinal immune homeostasis following dietary supplementation with Astragalus crude extract. Polysaccharides and flavonoids are likely among the key bioactive constituents contributing to these beneficial effects [29].

Several studies have convincingly demonstrated that oxidative stress occurs in weaned piglets regardless of weaning age [30]. The intestine is highly susceptible to oxidative stress, which promotes the excessive production of reactive oxygen species (ROS) and disrupts intestinal redox homeostasis and physiological function [31]. The endogenous antioxidant defense system, comprising enzymes such as SOD, CAT, and GPX, works cooperatively to eliminate superoxide radicals and hydrogen peroxide, thereby preventing ROS accumulation and mitigating oxidative stress [32]. In vitro studies have confirmed that flavonoids and polysaccharides from Astragalus have strong free radical scavenging capacities, including the neutralization of DPPH and hydroxyl radical (OH•) [33,34]. Previous study has shown that dietary Astragalus extract and *Codonopsis Pilosulae* blends supplementation increased serum T-AOC level and SOD activity in weaned piglets [28]. Moreover, Astragalus polysaccharides have also been reported to alleviate the decrease in serum T-SOD and T-AOC levels in weaned piglets caused by lipopolysaccharide [12]. In line with these findings, our results demonstrated that Astragalus crude extract addition significantly enhanced intestinal T-AOC and reduced MDA levels in weaned piglets, further demonstrating its antioxidant efficacy. The Nrf2 signaling pathway plays a crucial role in regulating cellular redox homeostasis by regulating the expression of antioxidant-related genes such as *HO-1* and *NQO1* [35]. Consistent with the observed improvements in antioxidant enzyme activities, this study also found that Astragalus crude extract supplementation upregulated the expression of key antioxidant-related genes (*SOD1*, *GPX1*, *Nrf2*, and *Trx*) in the intestinal tissues of piglets. This finding aligns with previous reports that Astragalus extract enhances antioxidant function in intestinal epithelial cells via activation of the Nrf2 signaling pathway [36]. Furthermore, inconsistent *CAT* mRNA expression in the jejunum and ileum was observed in the present study. And Chen et al. [37] showed that lipopolysaccharide treatment significantly decreased *CAT* and *Cu/Zn-SOD* expressions in the jejunal mucosa but increased these expressions in the ileum mucosa. We think this difference may be due to the different responses of different intestinal segments. However, this difference needs further investigation. Collectively, these findings suggest that Astragalus crude extract enhances antioxidant capacity in weaned piglets primarily by reinforcing enzymatic antioxidant systems and modulating the expression of antioxidant genes, which may represent a potential mechanism underlying the reduced diarrhea incidence and improved growth performance observed in this study.

The intestinal barrier serves as the primary defense against pathogens and toxins, playing a crucial role in maintaining intestinal homeostasis [38]. Weaning stress-induced intestinal inflammation and oxidative stress further lead to increased intestinal permeability and impaired barrier function. In the present study, supplementation with either Astragalus crude extract or antibiotics effectively upregulated the expression of occludin and CLDN1 proteins, two key tight junction proteins, suggesting a protective effect on mucosal integrity during the vulnerable weaning transition. These findings align with previous reports on Astragalus-derived bioactive constituents. For instance, Astragaloside IV has been shown to enhance the expression of TJ proteins such as ZO-1 and occludin in murine models of sepsis-induced intestinal injury [39]. Similarly, Astragalus polysaccharides have been reported to restore intestinal epithelium integrity by up-regulating tight junction proteins in radiation-induced gut damage, and have also been shown to increase the expression of occludin and claudin in lipopolysaccharide-induced weaned piglets [12,40]. The barrier-strengthening effects observed in this study may thus result from the synergistic action of multiple bioactive compounds present in the Astragalus crude extract. Moreover, plasma DAO activity is a sensitive biomarker of intestinal epithelium damage [41]. Consistent with the upregulation of tight junction proteins, both Astragalus crude extract and antibiotic supplementation significantly reduced plasma DAO activity, providing additional evidence for improved intestinal barrier integrity in weaned piglets. Notably, the barrier protection effect of Astragalus crude extract was comparable to that of antibiotic treatment. Overall, these results indicate that Astragalus crude extracts exhibit considerable potential to enhance barrier function and mitigate intestinal damage induced by weaning, supporting its viability as a functional alternative to antibiotics.

The gut microbiota constitutes an essential component of the intestinal barrier [42]. Dysbiosis of the gut microbiota, particularly an increase in harmful bacteria, leads to the production of more toxic metabolites, thereby compromising intestinal and overall host health [43]. In the present study, dietary Astragalus crude extract supplementation significantly altered the composition of the ileal microbiota in weaned piglets, although there was no significant difference in microbiota diversity among all groups. In contrast, antibiotic treatment exhibited a trend toward reduced microbial diversity and significantly altered the microbial composition. Notably, the CE group enriched several beneficial taxa, including *Lactobacillus*, *Clostridium*, *Blautia*, *Agathobacter,* and *Turicibacter*, while reducing the relative abundance of the potentially pathogenic *Streptococcus*. These findings align with previous reports that phytogenic supplements, such as berberine, can suppress the *Streptococcus* and increase beneficial genera such as *Agathobacter*, *Pediococcus*, *and Clostridium* in piglets [44]. *Agathobacter*, for instance, is a butyrate-producing bacterium, known to play a crucial role in maintaining intestinal epithelial barrier function and redox homeostasis [45], and its increase has been similarly observed in piglets receiving other herbal extracts *Piper sarmentosum* and guava leaves [46]. *Blautia*, a major acetate-producing genus with established probiotic properties [47], was positively associated with ileal T-AOC activity and plasma IgA content, while exhibiting a negative association with ileal mucosal MDA level in our research. This is consistent with a previous report that *Blautia* abundance was negatively correlated with oxidative stress markers such as serum MDA [48]. Furthermore, the genus *Turicibacter*, which is involved in bile acid and lipid metabolism [49], was also increased in the CE group and was significantly associated with elevated plasma IgA and reduced MDA levels, suggesting a potential association with immunomodulatory and antioxidant parameters. Previous studies have shown that natural polysaccharide fucoidan can enrich *Turicibacter* and ameliorate intestinal inflammation in colitis mouse models [50]. Collectively, our results indicate that CE beneficially modulates the gut microbiota, particularly enriching bacterial taxa which possess antioxidant and immunomodulatory functions. These microbiota-mediated improvements likely contribute to the observed enhancement of intestinal barrier function, reduction in diarrhea incidence, and overall improvement in growth performance. However, the precise mechanisms underlying these associations remain to be elucidated and require further validation through fecal microbiota transplantation.

## 5. Conclusions

In summary, these findings suggest that dietary supplementation with Astragalus crude extract could enhance the growth performance of weaned piglets by maintaining intestinal homeostasis, improving antioxidant status, and modulating gut microbiota structure and composition. Notably, Astragalus crude extract exhibited growth-promoting properties similar to those observed with antibiotic treatment, highlighting its potential as a natural alternative to conventional antibiotics. However, this study evaluated only a single dose of Astragalus crude extract, which represents a limitation and warrants further investigation to determine the optimal dosage for efficient application in piglets. In addition, although we have preliminarily proposed the potential synergistic interactions among the bioactive components of Astragalus crude extract, this hypothesis remains to be validated through comparative studies using purified individual components and further investigated using approaches such as network pharmacology.

## Figures and Tables

**Table 1:** Ingredient composition and nutrient levels in the basal diet (as-fed basis, %).

Item	Day 0–14	Day 15–28
Ingredients		
Corn	48.83	52.23
Soybean meal	18.00	15.00
Extruded soybean	13.00	10.75
Whey powder	5.00	3.22
Fish meal	2.62	2.82
Wheat bran	6.00	10.00
Dicalcium phosphate	1.50	1.12
L-Lys HCl	0.50	0.38
DL-Met	0.25	0.18
Soybean oil	2.00	2.00
Salt	0.30	0.30
Premix ^1^	2.00	2.00
Total	100.00	100.00
Nutrient levels ^2^		
Digestible energy, MJ/kg	13.24	12.89
Crude protein	19.58	18.90
Calcium	0.73	0.62
Nonphytate phosphorus	0.53	0.47
Methionine + cysteine	0.68	0.64
Lysine	1.38	1.05
Threonine	0.78	0.63
Tryptophan	0.25	0.20

^1^ Vitamin–mineral premix provided the following per 1 kg of complete diet: vitamin A 13,500 IU, vitamin D3 3500 IU, vitamin E 45 IU, vitamin K3 13 mg, vitamin B1 35 mg, vitamin B2 30 mg, vitamin B6 40 mg, vitamin B12 200 μg, nicotinic acid 60 mg, folic acid 2.4 mg, biotin 0.27 mg, D-pantothenic acid 24 mg; Cu (CuSO_4_·5H_2_O) 30 mg, Fe (FeSO_4_·H_2_O) 250 mg, Mn (MnSO_4_·5H_2_O) 40 mg, Zn (ZnCl_2_·5H_2_O) 110 mg, Se (Na_2_SeO_3_·H_2_O) 0.75 mg, I (Ca(IO_3_)_2_) 0.8 mg, NaCl 20 g. ^2^ The nutrient levels were calculated values.

**Table 2:** Effect of AN or CE on the growth performance and diarrhea incidence in weaned piglets.

Item	CON	AN	CE	SEM	*p*-Value
Body weight, kg					
Day 0	8.15	8.14	8.12	0.048	0.968
Day 14	11.83 ^b^	13.50 ^a^	13.31 ^a^	0.277	0.001
Day 28	17.48 ^b^	20.06 ^a^	19.69 ^a^	0.429	0.002
Average daily gain (g/d)					
Days 0–14	266.90 ^b^	382.87 ^a^	369.77 ^a^	18.732	<0.001
Days 15–28	403.80 ^b^	468.33 ^a^	455.93 ^ab^	12.839	0.032
Days 0–28	335.37 ^b^	425.57 ^a^	412.77 ^a^	14.983	0.002
Average daily feed intake (g/d)					
Days 0–14	502.23	552.83	540.88	10.999	0.065
Days 15–28	700.24 ^c^	814.32 ^a^	762.96 ^b^	16.769	<0.001
Days 0–28	596.24 ^c^	719.55 ^a^	684.99 ^b^	18.848	<0.001
Feed/gain					
Days 0–14	1.88 ^a^	1.45 ^b^	1.46 ^b^	0.078	0.004
Days 15–28	1.75	1.74	1.68	0.039	0.799
Days 0–28	1.78	1.69	1.66	0.036	0.367
Diarrhea index (days 0–14)					
Diarrhea rate, %	2.17 ^a^	1.34 ^b^	1.37 ^b^	0.167	0.043
Fecal score	2.56 ^b^	2.96 ^a^	2.89 ^ab^	0.077	0.046

CON, a control fed the basal diet; AN, the basal diet with 1000 mg/kg aureomycin; CE, the basal diet containing 500 mg/kg Astragalus crude extract. SEM, standard error of the means. ^a^, ^b^, ^c^ within a row, values with no common superscripts differ significantly (*p* < 0.05) between piglet feeding additives.

**Table 3:** Effect of AN or CE on apparent digestibility of dietary nutrients in piglets at 14 days post-weaning.

Item	CON	AN	CE	SEM	*p*-Value
Crude protein, %	75.36	77.43	81.95	1.483	0.083
Dry matter, %	86.85	86.16	87.96	0.738	0.667
Ether extract, %	53.92 ^b^	66.90 ^a^	58.26 ^ab^	2.388	0.047

CON, a control fed the basal diet; AN, the basal diet with 1000 mg/kg aureomycin; CE, the basal diet containing 500 mg/kg Astragalus crude extract. SEM, standard error of the means. ^a, b^ within a row, values with no common superscripts differ significantly (*p* < 0.05).

**Table 4:** Effect of AN or CE on plasma biochemistry in piglets at 14 days post-weaning.

Item	CON	AN	CE	SEM	*p*-Value
TP, g/L	42.13	43.08	42.61	0.391	0.675
ALB, g/L	28.96 ^b^	31.71 ^a^	30.37 ^b^	0.553	0.045
BUN, mg/dL	3.62 ^a^	1.93 ^c^	2.87 ^ab^	0.277	0.011
HDL-C, mmol/L	0.77	0.74	0.82	0.024	0.401
LDL-C, mmol/L	1.17 ^a^	0.85 ^b^	0.91 ^b^	0.060	0.029
TC, mmol/L	2.08 ^a^	1.63 ^b^	1.73 ^b^	0.078	0.015
TG, mmol/L	0.33	0.30	0.36	0.015	0.324
AST, U/L	57.96	71.00	52.59	4.754	0.300
ALT, U/L	63.47	69.15	69.25	5.259	0.893
ALP, U/L	27.36	34.71	12.87	5.333	0.260

CON, a control fed the basal diet; AN, the basal diet with 1000 mg/kg aureomycin; CE, the basal diet containing 500 mg/kg Astragalus crude extract. TP, total protein; ALB, albumin; BUN, blood urea nitrogen; ALP, alkaline phosphatase; AST, aspartate aminotransferase; ALT, Alanine aminotransferase; HDL-C, high-density lipoprotein cholesterol; LDL-C, low-density lipoprotein cholesterol; TG, triglyceride; TC, total cholesterol. SEM, standard error of the means. ^a, b, c^ within a row, values with no common superscripts differ significantly (*p* < 0.05).

## Data Availability

The datasets presented in this study are available in the repository of the National Center for Biotechnology Information under accession number PRJNA1414305 (https://www.ncbi.nlm.nih.gov/).

## Supplementary Materials

The following supporting information can be downloaded at: https://www.mdpi.com/article/10.3390/vetsci13030242/s1. Table S1: Ingredient composition and nutrient levels in the basal diet (as-fed basis, %). Table S2: Primers are required for real-time fluorescence quantitative PCR.
