# Supplementary material for: Dietary Astragalus Crude Extract Boosts Piglet Growth and Mitigates Weaning Stress by Enhancing Antioxidant Activity, Improving Immunity, and Modulating Intestinal Microbiota
Source: Vet Sci. 2026 Mar 3;13(3):242. doi: 10.3390/vetsci13030242 (PMC13029824; doi:10.3390/vetsci13030242)
Supplement: Supplementary file 1 [file vetsci-13-00242-s001.zip › vetsci-4150930-supplementary.pdf]

Table S1 Ingredient composition and nutrient levels in the basal diet (as-fed basis, %)

**Item**	**Day 0-14**	**Day 15-28**
**Ingredients**		
Corn	48.83	52.23
Soybean meal	18.00	15.00
Extruded soybean	13.00	10.75
Whey powder	5.00	3.22
Fish meal	2.62	2.82
Wheat bran	6.00	10.00
Dicalcium phosphate	1.50	1.12
L-Lys · HCl	0.50	0.38
DL-Met	0.25	0.18
Soybean oil	2.00	2.00
Salt	0.30	0.30
Premix[1]	2.00	2.00
Total	100.00	100.00
**Nutrient levels[2]**		
Digestible energy, MJ/kg	13.24	12.89
Crud protein	19.58	18.90
Calcium	0.73	0.62
Nonphytate phosphorus	0.53	0.47
Methionine + cysteine	0.68	0.64
Lysine	1.38	1.05
Threonine	0.78	0.63
Tryptophan	0.25	0.20

[1] Vitamin-mineral premix provided the following per 1 kg of completed diet: vitamin A 13,500 IU, vitamin D3 3,500 IU, vitamin E 45 IU, vitamin K3 13 mg, vitamin B1 35 mg, vitamin B2 30 mg, vitamin B6 40 mg, vitamin B12 200 μg, nicotinic acid 60 mg, folic acid 2.4 mg, biotin 0.27 mg, D-pantothenic acid 24 mg; Cu ($CuSO_4 \cdot 5H_2O$) 30 mg, Fe ($FeSO_4 \cdot H_2O$) 250 mg, Mn ($MnSO_4 \cdot 5H_2O$) 40 mg, Zn ($ZnCl_2 \cdot 5H_2O$) 110 mg, Se ($Na_2SeO_3 \cdot H_2O$) 0.75 mg, I ($Ca(IO_3)_2$) 0.8 mg, NaCl 20 g.

[2] The levels of crude protein, calcium, and phosphorus were analyzed values; others were calculated values.

Table S2 Primers are required for real-time fluorescence quantitative PCR

Genes	Primer (5′→3′)	Product size, bp	Accession number
SOD1	F: GCAGGTCCTCACTTCAATCC<br>R: CTTCCAGCATTTCCCGTCTT	248	NM_001190422
CAT	F: CACACATACCCATTCGTCACT<br>R: CAGCCCTAACCTTCACTTACC	157	NM_214301
NQO1	F: AGTATCCTGCCGAGACTGCTCTG<br>R: CACAAGGTCTGCGGCTTCCAC	95	NM_001159613
HO-1	F: GTTTGAGGAGGTGCAGGAGC<br>R: GAGTGTCAGGACCCATCGGA	184	NM_001004027.1
GPX1	F: CGATGCCACTGCCCTCAT<br>R: GGCCCACCAGGAACTTCTC	98	NM_214201
Nrf2	F: CCCATTCACAAAAGACAAACATTC<br>R: GCTTTTGCCCTTAGCTCATCTC	72	XM_005671981.3
Trx	F: TTCCAATGTCGTGTTCCTTG<br>R: ACCCACCTTCTGTCCCTTTT	115	NM_214313.2
GAPDH	F: CCTGTTGCTGTAGCCAAATTC<br>R: GCTACACTGAGGACCAGGTTG	146	XM_021091114.1

SOD1, superoxide dismutase 1; *CAT*, catalase; *NQO1*, NAD(P)H quinone dehydrogenase 1; *HO-1*, heme oxygenase; *GPX1*, glutathione peroxidase 1; *Nrf2*, nuclear factor erythroid 2-related factor 2; *Trx*, thioredoxin.
